# Chronic Exposure to Oral Pathogens and Autoimmune Reactivity in Acute Coronary Atherothrombosis

**DOI:** 10.1155/2014/613157

**Published:** 2014-02-25

**Authors:** Ivana Burazor, Aristo Vojdani

**Affiliations:** ^1^Cardiology Department, Institute for Rehabilitation, Sokobanjska 17, 11000 Belgrade, Serbia; ^2^Immunosciences Lab Inc, Los Angeles, CA 90035, USA

## Abstract

*Background*. It has been hypothesized that various infective agents may activate immune reactions as part of the atherosclerotic process. We aimed to investigate the interrelationship between chronic exposure to oral pathogens and immune-inflammatory response in patients with acute coronary atherothrombosis. *Patients and Methods*. The study included 200 participants from Serbia: 100 patients with acute myocardial infarction (MI), and 100 age- and sex-matched controls. Antibodies to oral anaerobes and aerobes were determined as well as autoantibodies to endothelial cells, beta-2 glycoprotein I, platelet glycoprotein IIb/IIIa and anticardiolipin. Interleukin-6 (IL-6) and C-reactive protein (CRP) were measured. *Results*. The mean serum antibodies to oral anaerobes tended to be higher among subjects with MI (0.876 ± 0.303 versus 0.685 ± 0.172 OD, *P* < 0.001). Similarly, antibody levels against oral aerobes in patients were significantly different from controls. Antibodies against endothelial cell, beta-2 glycoprotein I, platelet glycoprotein IIb/IIIa, anticardiolipin along with CRP and IL-6 were highly elevated in patients. The levels of antibodies to oral bacteria showed linear correlation with tissue antibodies, CRP and IL-6. *Conclusion*. Antibody response to chronic oral bacterial infections and host immune response against them may be responsible for the elevation of tissue antibodies and biomarkers of inflammation which are involved in acute coronary thrombosis development.

## 1. Introduction 

It has been recently hypothesized that various infectious diseases, both bacterial and viral, may activate vessel-associated leucocytes or immune reactions in the atherosclerotic process. Studies have also shown a strong association between poor dental health and cardiovascular diseases [1–3]. There are circumstances in which the presence or absence of teeth and the bacteria that reside on them could be the risk factors for the triggering of cerebrovascular and cardiovascular disorders, such as myocardial infarction. Several hypotheses can explain this scenario, among which asymptomatic bacteremia might play a role. Our body surface is colonized by over 10^12^ bacteria. A minuscule proportion of these bacteria gain access to our underlying tissue and are quickly dispatched by the body's immune response [4–7]. The bacteria, typically *Streptococcus sanguis, Streptococcus oralis, *and *Peptostreptococcus anaerobius,* arise in the oral cavity and are believed to enter the bloodstream as a result of trauma as bland as the manipulations of oral hygiene [8]. These bacteremias may infect sites of underlying pathologic changes of heart valves [9, 10]. On the damaged heart valves, adherent bacteria soon become embedded and protected in newly formed thrombi or platelet vegetation. Consequently, streptococci capable of initial adhesion and rapid induction of thrombosis are likely to be more virulent in clinical disease. As many as half of all cases of bacterial endocarditis have been attributed to viridans streptococci, with *S. sanguis* identified as the vector three to four times more frequently than *S. oralis* [11, 12]. This association may reflect the large proportion of these microorganisms in the oral flora and the frequency of these bacteremias in comparison with those that arise from other organs and tissues. The specificity of infection may also reflect special virulence traits of these bacteria. *Porphyromonas gingivalis, Prevotella intermedia, *and* Bacteroides forsythus* are Gram-negative small basil quality obligate anaerobic bacteria and are held directly responsible for the formation of periodontitis. These bacteria usually secrete brown-black pigments and form colonies when they reproduce in blood agar plates used for their cultivation. These bacteria were classified in the *Bacteroides* genus until 1988 and 1990, when they were reclassified to the *Porphyromonas* and *Prevotella* genera, respectively, in accordance with new classification strategies made by Shah and Collins [13, 14].

These anaerobic bacteria, in conjunction with the facultative anaerobic bacteria such as *Streptococcus* mentioned above, can lead to mixed types of infections affecting various tissues, including the joints and the heart [15–20]. An extensive number of virulence factors include fimbriae, degradative enzymes, exopolysaccharide capsules, and atypical lipopolysaccharides; these factors, through various mechanisms of action, including mimicry or citrullination of self-peptide, can induce inflammation and autoimmunity against various tissue antigens [21–23].

For example, immunological mapping using a library of cyclic citrullinated *α*-enolase peptides led to the identification of a B-cell-dominant epitope comprising amino acids 5-21 of *α*-enolase (KIHAREIFDSRGNPTVE) where arginine-9 and arginine-15 are citrullinated, with an 82% sequence similarity with that of *P. gingivalis* [24]. Immunization with citrullinated human and *P. gingivalis α*-enolase and citrullinated fibrinogen causes similar pathology in humanized DR4 transgenic mice. This mechanism may be triggered by the release of different cytokines and prostanoids, such as interleukin-1 (IL-1), IL-6, IL-8, tumor necrosis factor-alpha (TNF-*α*), prostaglandin E2, and different matrix metalloproteinases (MMP). These bacteria and released metabolites beyond this potential local pathogenicity may disseminate systemically and influence directly or indirectly the atheroma pathophysiology. Aside from increasing cytokine production, Gram-negative bacteria may also stimulate hypercoagulability, monocyte activation, and liver activation by releasing acute phase proteins, such as high-sensitivity C-reactive protein (hs-CRP) [4]. This repeated systemic exposure to orally derived bacteria, bacterial endotoxins, and systemic inflammation would eventually directly and/or indirectly affect the vascular walls, inducing a state of endothelial dysfunction.

The purpose of this study, then, is to investigate the potential interrelationship between chronic exposure to oral pathogens, the antibodies produced against them, and elevations in the levels of markers of immune-inflammatory response in patients with acute myocardial atherothrombosis.

## 2. Materials and Methods

### 2.1. Study Population

The study comprised 200 participants from Serbia, of whom 100 were patients admitted due to acute myocardial infarction. 100 were age- and sex-matched controls. In the patients group, the diagnosis of acute myocardial infarction was based on evidence of myocardial necrosis in a clinical setting consistent with acute myocardial ischemia [25]. The inclusion criteria for control groups were absence of known coronary artery diseases (previous stable or unstable angina as well as previous myocardial infarction) or carotid disease; initial electrocardiography (ECG) was recorded to confirm the absence of coronary artery disease. The exclusion criteria for the group of patients and also for controls were concomitant dilated cardiomyopathy, valvular heart disease, atrial fibrillation, major surgery, or trauma within previous months. All patients and controls with known or suspected thrombotic disorders, systemic illness, autoimmune diseases, sepsis, alcohol liver diseases, chronic obstructive pulmonary diseases, acute respiratory infections, current infections of any etiology or infections within previous 3 weeks, and malignancy and inflammatory diseases were also excluded. Study participants were asked about the risk factors for coronary artery disease (CAD), that is, smoking status, family history of CAD, hypertension, dyslipidemia, and diabetes.

The majority of the study participants were males (60% of patients and 58% of controls, *P* = n.s). The mean age of patients was 59.42 years and 59.03 years in controls (*P* = n.s).

The investigation conformed to the principles outlined in the Declaration of Helsinki. Signed informed consent or witnessed oral informed consent was obtained from all patients and healthy controls in accordance with the guidelines of the Ethical Review Committee of the Medical Faculty University of Nis, who approved the study protocol.

### 2.2. Preparation of Bacterial Antigens

Oral aerobes or facultative anaerobes (*Streptococcus sanguis*, *Streptococcus oralis*, and *Peptostreptococcus anaerobius*) and oral obligate anaerobes (*Porphyromonas gingivalis*, *Prevotella intermedia*, and *Bacteroides forsythus*) were purchased from American Type Culture Collection (Rockville, Maryland, USA) and cultivated according to the methods described earlier [8, 9]. In brief, bacteria were grown in different media and the cultures were incubated for 48–72 h at 35–37°C. Purity was assessed by colony morphology and gram stain. Bacteria were harvested at the late log phase by centrifugation at 10,000 g for 15 min and then washed twice with 0.15 M sodium chloride. The bacteria were lysed using a sonicator, and after separation of the lysate, the protein concentration was measured and used for coating ELISA plates and antibody measurement.

### 2.3. Serum Antibody Assay by Enzyme-Linked Immunosorbent Assay (ELISA)

Pathogen-specific antibody was quantitated by enzyme-linked immunoassay. Wells of microtiter plates were coated with 100 *μ*L of bacterial antigens (concentration of 10 *μ*g/mL in 0.1 M of carbonate buffer, pH 9.6). Plates were incubated overnight at 4°C and then washed three times with 200 *μ*L Tris-buffered saline (TBS) containing 0.05% Tween 20, pH 7.4. The nonspecific binding of immunoglobulins was prevented by adding 2% bovine serum albumin (BSA) to phosphate-buffered saline (PBS) and incubated overnight at 4°C.

Plates were washed as described above, and then serum samples diluted 1 : 200 in 0.1 M PBS Tween containing 2% BSA were added to duplicate wells and incubated for 1 h at room temperature. Plates were washed, and then alkaline phosphatase goat anti-human IgG F(ab′)2 fragments (KPI, Gaithersburg, MD) with optimal dilution of 1 : 400 in serum diluent were added to each well; plates were incubated for an additional 1 h at room temperature. After washing five times with TBS-Tween buffer, the enzyme reaction was started by adding 100 *μ*L of paranitrophenylphosphate in 0.1 mL diethanolamine buffer 1 mg/mL containing 1 mM MgCl_2_ and sodium azide pH 9.8. The reaction was stopped 45 min later with 50 *μ*L of 1 N NaOH. The optical density (OD) was read at 405 nm by means of a microtiter reader. To detect nonspecific binding, several control wells contained all reagents except human serum, or wells were coated with HSA followed by the addition of human serum and all other reagents to be used for specificity of the antigen-antibody reaction.

### 2.4. Autoimmunity and Immunity Markers

Antibodies against beta-2 glycoprotein I (IgG) were determined by using ELISA method (Calbiochem, La Jolla, CA, USA, Cat# B59407). Anticardiolipin antibodies were determined by using a test kit from Sigma, Cat# P1867. Antiendothelial cell, beta-2 glycoprotein I, and antiplatelet glycoprotein IIb/IIIa (IgG) antibodies were measured by coating each ELISA well plate with one *μ*g of pure antigen followed by the addition of serum. All additional steps are described in the ELISA section.

### 2.5. Markers of Inflammation

Levels of IL-6 and hs-CRP were measured using kits manufactured by Diagnostic Products Corporation, Los Angeles, CA, on an IMMULITE Automated Immunoassay Analyzer. The IMMULITE system utilizes assay-specific, antibody- or antigen-coated plastic beads as the solid phase, alkaline phosphatase labeled reagent, and a chemiluminescent substrate. The IMMULITE system automates the entire assay process. Light emission was measured by a photomultiplier tube, and the results were calculated for each sample using different calibrators and controls.

The established reference ranges of the lab performing the tests were from 0.7 to 4.6 pg/mL for IL-6 and from 0 to 1 mg/dL for hs-CRP. Values above the established reference ranges were marked as positive.

### 2.6. Statistical Analysis

Results of normally distributed continuous variables are expressed as the mean value ± standard deviation. Analysis of normality of the continuous variables was performed with the Kolmogorov-Smirnov test. Differences between examined groups were assessed by unpaired *t*-test and Mann-Whitney *U* test and *χ* ^2^ testing was used for discrete variables. Relative Risk (RR), odds ratio (OR), and 95% Confidence Interval (CI) for the RR and OR were calculated.

Correlations between continuous variables were analyzed with the two-way Pearson correlation tests. Hs-CRP levels were not of linear nature. Therefore, in order to fulfill the statistical requirement, hs-CRP was logarithmically transformed before entering the analysis. Differences were considered to be significantly important if the null hypothesis could be rejected with > 95% confidence. All *P* values were two-tailed. The PASW 18.0 statistical software package was used for all calculations.

## 3. Results

### 3.1. Clinical Characteristics of Patients with Acute Myocardial Infarction

In this study, we measured the levels of antibodies against oral pathogens as well as antibodies against endothelial cells, beta-2 glycoprotein I, platelet glycoprotein IIb/IIIa, anticardiolipin antibodies, and inflammatory markers such as hs-CRP and interleukin 6, in blood samples of patients with myocardial infarction and compared them to the levels of the same antibodies and markers in samples from control subjects.

Table 1 outlines clinical characteristics of patients with acute myocardial infarction. Mean value of systolic blood pressure was 132 ± 35.98 mm Hg. Patients spent in hospital a period of 10.8 ± 5.4 days to fulfill medical treatment.

### 3.2. Antibodies against Oral Pathogens

**Figure 1:**
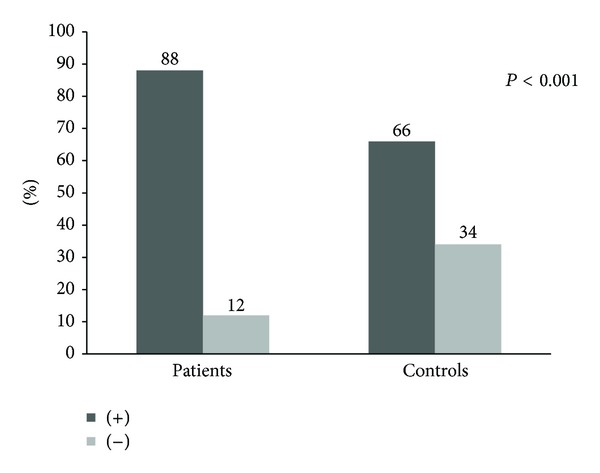
Comparison of the levels of IgG antibodies against oral anaerobes within the study participants.

IgG antibodies to oral anaerobes were highly present in patients with acute coronary atherothrombosis. A total of 88% of patients with cardiovascular disease had elevated antibodies above the mean of controls, as shown in Figure 1; RR was 1.33 (1.13 to 1.56) 95% CI.

Overall, the mean OD of serum IgG antibodies to oral anaerobes tends to be higher among subjects with coronary artery disease than those without (0.876 ± 0.303 OD versus 0.685 ± 0.172, *P* < 0.001) (Table 2). 

**Figure 2:**
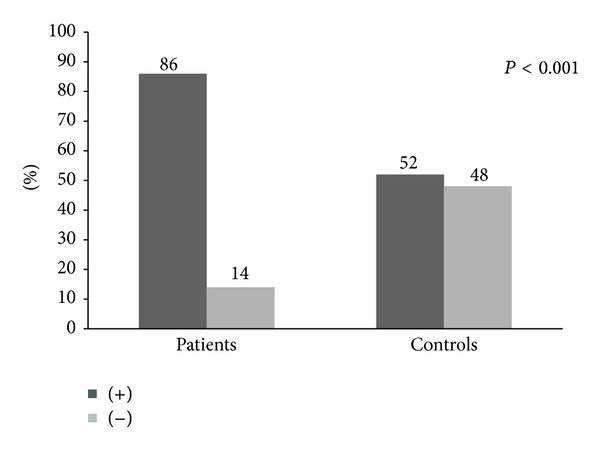
Comparison of the levels of IgG antibodies against oral aerobes within the study participants.

IgG antibodies to oral aerobes were highly present in patients with acute coronary atherothrombosis. A total of 86% of patients had antibodies detectable compared to 52% of controls, RR 1.65 (1.34 to 2.02; 95% CI) (Figure 2).

The mean OD of serum IgG antibodies to oral aerobes tends to be higher among subjects with coronary artery disease than those without (0.996 ± 0.323 OD versus 0.769 ± 0.239 OD and *P* < 0.001) (Table 2).

### 3.3. Autoimmunity and Inflammation

**Figure 3:**
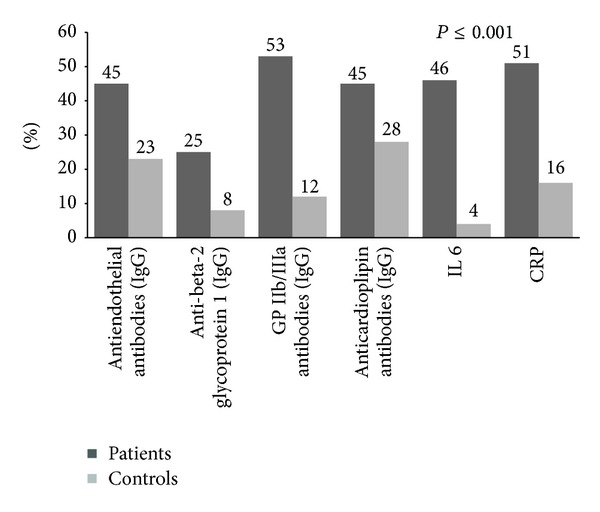
Comparison of the levels of IgG antibodies against antigens associated with autoimmunity activation and markers of inflammation in the study participants.

Subjects with acute coronary artery atherothrombosis showed very strong autoimmune response with elevation in antiendothelial cell IgG antibodies in the group (45% versus 23%, O.R. 2.73, 95% CI for OR 1.48–5.04, RR 1.95, 95% CI for RR 1.28–2.97, *χ* ^2^ = 3.14, *P* = 0.001, Figure 3.

The mean serum antiendothelial cells IgG antibodies were 0.684 ± 0.211 OD in patients versus 0.598 ± 0.193 OD in controls, *P* = 0.004 (Table 3).

Also, anti-beta-2 glycoprotein I antibodies IgG were detected in 25% of patients with acute coronary atherothrombosis compared to 8% of controls (OR 3.91, 95% CI 1.67–9.18, RR 3.12, 95% CI for RR 1.48–6.59, *χ* ^2^ = 2.992, *P* < 0.001); see Figure 3. The mean serum titers are shown in Table 3.

Antibodies to platelet glycoprotein IIb/IIIa were detected in 53% of patients and 12% of controls (OR 8.26, 95% CI 4.02–16.98, RR 4.41, 95% CI for RR 2.51–7.74, *χ* ^2^ = 5.18, *P* < 0.001), Figure 3.

Anticardiolipin antibodies were detected in 45% of patients and 28% of controls (OR 2.10, 95% CI for OR 1.16–3.78; RR 1.6, 95% CI for RR 1.09–2.35, *χ* ^2^ = 2.43, *P* < 0.001). The titers were significantly different between groups, as shown in Table 3.

Our study showed that 46% of patients had elevated levels of circulating IL-6. Statistically, this proportion is significantly higher compared to controls (only 4%), (*χ* ^2^ = 5.53, *P* < 0.001; odds ratio (OR), 20.44; (95% CI, 6.57–59.88); RR 11.5 (4.3 to 30.7). Concentrations of IL-6 were significantly higher in patients compared to controls (9.38 pg/mL (2.00–18.85) versus 1.5 pg/mL (1.2–1.8), *P* < 0.001).

There was a significant difference between patients and controls in regard to CRP; 51% of patients had CRP above reference range compared to 16% of controls, *χ* ^2^ = 4.65, *P* < 0.001, RR 3.18 (1.95–5.49): OR 5.46 (2.81–10.63) (Figure 3). The median of this marker of inflammation was 2.67 mg/dL (0.384–20.895) in patients and 0.225 mg/dL (0.075–0.623) in controls, *P* < 0.001.

Antibodies to both oral anaerobes and aerobes showed strong and significant correlation with different parameters of autoimmunity, immunity, and inflammation. Pearson's linear correlation, coefficient of correlation (*r*), and *P* values are shown in Tables 4 and 5.

## 4. Discussion

Our understanding of the pathogenesis of the acute thrombotic complications of the atherosclerosis has burgeoned in recent years. We now understand that many acute thrombotic coronary occlusions do not necessarily result from critically stenosed sites in the arteries. This distinction between lesions versus lumen diameter challenges our traditional reliance upon coronary anatomy [26–29]. Atherothrombosis is the major determinant of acute ischemic cardiovascular events, such as myocardial infarction and stroke. Thus, its understanding is essential to enable the development of targeted and more effective therapies. Although related in part to alterations in lipid metabolism, atherosclerosis is now considered a primarily immune-mediated disease [30].

The role of the immune system and autoimmune reactions in atherosclerosis appears to be a double edged-sword, with some of them being proatherogenic, while others can be antiatherogenic depending on what stage in the long-lasting process of atherosclerosis.

The purpose of our study, then, was to investigate the potential interrelationship between chronic exposure to oral pathogens, the antibodies produced against them, and elevations in the levels of markers of immune-inflammatory response in acute, urgent, and lifesaving clinical settings in patients with acute myocardial atherothrombosis.

Our results indicated that IgG antibodies against oral pathogens (oral aerobes/facultative anaerobes) and oral obligate anaerobes were highly present in the patients with acute myocardial infarction, suggesting high exposure to chronic infection. Upon searching the literature, we found that it is has been recently proposed that chronic infections (bacterial *Helicobacter pylori*, *Chlamydia pneumonia,* and periodontitis among many others) can contribute to the development of atheromas either directly (endothelial injury, invasion of endothelial cells, and platelet aggregation) or indirectly (production of antibodies to lipopolysaccharide, cytokines and dysfunction of the immune system) [31].

In response to infection (e.g., oral bacteria among others), the immune system jumps into action, deploying cells as well as antibodies in order to recognize and destroy the invaders. Antibodies are molecules produced by plasma cells and B cells against the “enemy”—the infectious agent. However, owing to molecular mimicry or antigenic similarity between these infectious agents and human tissue structure, in a genetically susceptible individual, components of the body's immune system target one or more types of the person's own tissue, which may result in autoimmunity [18, 32, 33].

Taking these together, evidence indicates that infectious agents play a pivotal role in the induction of autoimmunities. The question of how infectious agents contribute to autoimmunity has continued to be of interest to clinical and basic researchers and immunologists in general [18].

In many cases, it is not a single infection but rather the “burden of infections” from childhood that is responsible for the induction of autoimmunity [18]. Thus, oral pathogens can also give their contribution towards autoimmunity.

An example of this is the case of anti-phospholipid (aPL) syndrome, in which anticardiolipin and anti-beta-2 glycoprotein I pathogenic antibodies are detected. In patients with systemic lupus erythematosus (SLE) or antiphospholipid syndrome, serum complexes and anti-beta-2 glycoprotein-I-oxidized-LDL complex autoantibodies are elevated [34, 35]. Similarly, such complexes and antibodies found in the bloodstream of patients with vascular complications, such as myocardial infarction and unstable angina, strongly associate with arterial thrombosis.

Our results indicated that anti-beta-2 glycoprotein antibodies and anticardiolipin antibodies (aCLs) can be detected in patients with myocardial infarction.

The data is similar from case-control studies that demonstrate the association of aCLs with stroke and acute myocardial infarction [32, 36]. Also, the authors found that IgG/IgM/IgA aCL and IgA for anti-beta-2 glycoprotein I associated with increased risk of ischemic stroke, arterial thrombosis, atherosclerotic immune process, acute myocardial infarction, and peripheral vascular diseases [36]. Although the exact mechanisms remain unknown, anti-beta-2 glycoprotein I was thought to interact with beta 2-glycoprotein I on the endothelial membrane and induce inflammatory reactions [37].

Artenjak et al. [38] reported on the correlation between aPL and cardiovascular risk in nonautoimmune settings. Taken together, these results did not demonstrate a clear association between aPL and acute cardiovascular events.

Beta-2-glycoprotein I is present at high concentrations in the blood stream and is expressed by many cell populations, including endothelial cells, lymphocytes, and monocytes. It binds negatively not only charged molecules, including phospholipids, heparin, and oxLDL, but also the surface of activated platelets and the membrane of apoptotic cells [39–43]. In our study population, those autoantibodies were highly present in patients with acute myocardial infarction. One should have in mind that antiendothelial cell antibodies may cause vasculitis as part of an autoimmune response. This is a heterogeneous family of antibodies. The IgG antibodies are highly present also in the blood sera of SLE patients and may mediate immunologic injury to blood vessel walls.

Finally, significant elevation in the levels of IL-6, CRP and endothelial cell antibody indicates that inflammation driven by oral pathogens plays a significant role in the development of atherothrombosis [44–48].

## 5. Conclusion

Taking together the above presented data, it appears that oral pathogens, through the release of toxins, seem to be capable of inducing changes in the host proteins. These can be recognized by the immune system, triggering an inflammatory process associated with the clinical manifestation of atherosclerosis—acute myocardial infarction. This and other immunopathogenic mechanisms need to be further elucidated.

## Figures and Tables

**Table 1:** Characteristics of patients with acute myocardial infarction.

Characteristics	(%)
ECG abnormalities at entry	
ST segment elevation	45
Without ST segment elevation	55
Systolic BP, mmHg	
<120	32.4
120–139	21.6
140–159	15.4
>160	30.6
Mean (SD)	132 ± 35.98
Diastolic BP, mean (SD), mmHg	79 ± 23.33
Heart rate, heartbeats/min	
<70	16.3
70–89	38.7
90–109	36.9
>110	8.1
Mean (SD), mmHg	22.73
Previous disease	
Previous MI	29.7
Previous CABG	17.1
Aspirin before admission	34.2
Duration of staying in hospital, mean (SD)	10.8 (5.4)
LVEF, mean (SD)	54.4 (13.30)
LVEF < 40	16 (16.2)
New event	19.6

ECG: electrocardiogram; BP: blood pressure; MI: myocardial infarction; CABG: coronary artery bypass grafting; LVEF: left ventricle ejection fraction. Values are % unless otherwise indicated.

**Table 2:** Antibodies against oral pathogens in the study participants.

ORAL pathogen (Bacterial agent)	Study participants	Mean	SD	95% CI		*P *
				Lower bound	Upper bound	
Oral anaerobes (OD)	Patients	0.876	0.303	0.662	1.035	<0.001
	Controls	0.685	0.172	0.569	0.715	

Oral aerobes (OD)	Patients	0.996	0.323	0.768	1.226	<0.001
	Controls	0.769	0.239	0.622	0.873	

**Table 3:** Autoantibodies in the study participants.

Autoantibodies	Group	Mean	S.d.	95% CI		Minimum	Maximum	*P *
				Lower bound	Upper bound			
Antiendothelial cells (OD)	Patients	0.684	0.211	0.509	0.579	0.229	0.955	0.004
	Controls	0.598	0.193	0.444	0.494	0.215	0.775	

Beta 2- glycoprotein I (OD)	Patients	0.665	0.344	0.595	0.1735	0.242	2.266	0.003
	Controls	0.540	0.205	0.499	0.582	0.280	1.437	

Platelet glycoprotein IIb/IIIa (OD)	Patients	0.351	0.100	0.331	0.372	0.225	0.718	0.001
	Controls	0.306	0.074	0.290	0.321	0.176	0.499	

Anticardiolipin (OD)	Patients	0.552	0.180	0.515	0.589	0.228	1.161	0.001
	Controls	0.415	0.097	0.396	0.435	0.237	0.704	

**Table 4:** Correlations of oral anaerobes IgG with different parameters of autoimmunity and inflammation.

Autoantibodies and inflammation	Antibodies to oral anaerobes (IgG)	
	*r*	*P*
Antiendothelial cells	0.541	0.01
Anti-beta 2 glycoprotein I	0.459	0.01
Antiplatelets glycoprotein IIb/IIIa	0.499	0.01
Anticardiolipin	0.647	0.01
Interleukin 6	0.199	0.01
hs C-reactive protein	0.229	0.01

*r*—coefficient of correlation.

**Table 5:** Correlations of oral aerobes IgG with different parameters of autoimmunity and inflammation.

Autoantibodies and inflammation	Antibodies to oral aerobes (IgG)	
	*r*	*P*
Antiendothelial cells	0.547	0.01
Anti-beta-2 glycoprotein I	0.443	0.01
Antiplatelets glycoprotein IIb/IIIa	0.546	0.01
Anticardiolipin	0.686	0.01
Interleukin 6	0.180	0.01
hs C-reactive protein	0.149	0.01

*r*—coefficient of correlation.
